# Next Generation Semiconductor Based Sequencing of the Donkey (*Equus asinus*) Genome Provided Comparative Sequence Data against the Horse Genome and a Few Millions of Single Nucleotide Polymorphisms

**DOI:** 10.1371/journal.pone.0131925

**Published:** 2015-07-07

**Authors:** Francesca Bertolini, Concetta Scimone, Claudia Geraci, Giuseppina Schiavo, Valerio Joe Utzeri, Vincenzo Chiofalo, Luca Fontanesi

**Affiliations:** 1 Department of Agricultural and Food Sciences, Division of Animal Sciences, University of Bologna, Viale Fanin 46, Bologna, Italy; 2 Department of Veterinary Sciences, Animal Production Unit, University of Messina, Polo Universitario dell'Annunziata, Messina, Italy; 3 Meat Research Consortium, Polo Universitario dell’Annunziata, Messina, Italy; Wageningen UR Livestock Research, NETHERLANDS

## Abstract

Few studies investigated the donkey (*Equus asinus*) at the whole genome level so far. Here, we sequenced the genome of two male donkeys using a next generation semiconductor based sequencing platform (the Ion Proton sequencer) and compared obtained sequence information with the available donkey draft genome (and its Illumina reads from which it was originated) and with the EquCab2.0 assembly of the horse genome. Moreover, the Ion Torrent Personal Genome Analyzer was used to sequence reduced representation libraries (RRL) obtained from a DNA pool including donkeys of different breeds (Grigio Siciliano, Ragusano and Martina Franca). The number of next generation sequencing reads aligned with the EquCab2.0 horse genome was larger than those aligned with the draft donkey genome. This was due to the larger N50 for contigs and scaffolds of the horse genome. Nucleotide divergence between *E*. *caballus* and *E*. *asinus* was estimated to be ~ 0.52-0.57%. Regions with low nucleotide divergence were identified in several autosomal chromosomes and in the whole chromosome X. These regions might be evolutionally important in equids. Comparing Y-chromosome regions we identified variants that could be useful to track donkey paternal lineages. Moreover, about 4.8 million of single nucleotide polymorphisms (SNPs) in the donkey genome were identified and annotated combining sequencing data from Ion Proton (whole genome sequencing) and Ion Torrent (RRL) runs with Illumina reads. A higher density of SNPs was present in regions homologous to horse chromosome 12, in which several studies reported a high frequency of copy number variants. The SNPs we identified constitute a first resource useful to describe variability at the population genomic level in *E*. *asinus* and to establish monitoring systems for the conservation of donkey genetic resources.

## Introduction

The donkey (*Equus asinus*) was probably domesticated by African pastoralists who started to breed Nubian (and possibly Somali) wild asses to cover their need to facilitate transportation and movements during a period of increasing aridity of the Sahara, about 6000 years ago [1–4]. Then, its domestication contributed to the expansion of overland trade in Africa and western Asia and the raise of the Egyptian state at that time [2, 3]. Since then, donkeys that were spread in Europe (starting about 4000 years ago) and subsequently in other continents, have been mainly employed as pack and working animals without any substantial changes in their use and without any particular improvements [5, 6]. It is estimated that about 95% of the current 43.5 million of donkeys in the world (mainly in developing countries and China) are kept specifically for work and for the production of other working animals, the hybrids with the horse: mules and hinnies [5, 7]. In Europe the number of donkeys is rapidly decreasing, due to the mechanization of the transports and agriculture, and many populations or breeds, generated mainly by geographical isolation, are highly endangered and close to the extinction or already extinct [6,8]. Economic interests in breeding donkeys have been recently raised for i) milk production to substitute vaccine milk for human consumption in case of allergies [9–13], ii) milk derived products for cosmetic applications [14], iii) meat production [15] and for iv) recreation and touristic activities [6].

Despite the relevance of this equid and the new raising interests around it, few studies investigated this species at the molecular genetic level (with DNA markers) or at the whole genome level (analyzing karyotypes and chromosome homologies). Most of these studies were oriented to describe genetic variability of different donkey breeds or populations for conservation purposes using microsatellite markers developed from the horse (*Equus caballus*), or using mitochondrial DNA to evaluate genetic differentiation and investigate the domestication processes of this species [16–23]. Other works were focused on a few relevant genes related to the emerging interests for the donkey (milk production) (i.e. [24, 25]) or related to the characterization of some populations (coat color) [26]. However, it is clear that most of the studies that investigated donkey genes or DNA markers can be considered, to some extent, byproducts or were linked to related investigations that started from the horse or included this latter species in some way, due to the more advanced research activities and genomic information available in *E*. *caballus* [27–29]. This is possible because the two species (*E*. *caballus* and *E*. *asinus*), despite the different number of chromosomes (2N = 64 and 62, respectively) and several other rearrangements identified by comparative mapping, chromosome painting and zoo-FISH [30–32], are closely related, as also demonstrated by the fact that crossing the two species, hybrids are viable (mules and hinnies) and in few cases are not completely sterile [33–35]. A recent re-estimation based on molecular data indicated that all *Equus* species diverged from a common ancestor about 4.0–4.5 million of years before present (Myr BP) [36]. The same study reported the first draft assembly of the donkey genome (based on short Illumina reads obtained from one donkey) that was used as outgroup in the phylogenetic analyses that recalibrated the evolution of the *Equus* genus [36]. Single nucleotide polymorphisms (SNPs) were also mined from these data [36]. However, as far as we know, thus far no other studies investigated the donkey genome to identify polymorphisms on a large scale.

Next generation semiconductor-based sequencing technology (Ion Torrent technology) has been recently proposed as a new potentially cost effective next generation sequencing platform with a rapid turnaround time [37, 38]. The technology is based on the detection of small modifications of pH (derived by production of H^+^) obtained during the DNA elongation steps and captured by a chip constructed to accommodate sequencing reactions [37]. The first sequencing platform (Ion Torrent Personal Genome Machine—PGM) has been largely applied for sequencing simple genomes (i.e. viruses, bacteria, mtDNA, etc.) or parts of them or for target re-sequencing of genes or genomic regions in complex genomes for a large number of applications focused on clinical, forensic and research investigations (i.e. [39–42]). The advent of a second machine using the same technology (Ion Proton) extended the range of applications of semiconductor sequencing, increasing the throughput of each run [43]. We already used the Ion Torrent PGM for SNP discovery and analysis in complex mammalian genomes using reduced representation libraries [44] generated for the rabbit and pig genomes or obtained by resequencing amplicons in candidate genes for production traits in pigs [45–47].

In this study we sequenced the genome of two donkeys using the Ion Proton sequencer and compared obtained sequence information with the available *E*. *asinus* draft genome [36] and with the EquCab2.0 assembly of the horse genome [27], for a comparative sequence data analysis between the two species (adding information from the already reported Illumina sequencing reads [36]). Then, we combined whole genome sequence data with Ion Torrent sequences obtained from a reduced representation library (RRL) generated from genomic DNA of donkeys of different breeds/populations for a first mass SNP discovery and description in *E*. *asinus*.

## Materials and Methods

### Ethical statement

Blood sampling was carried out by experienced veterinarians for health monitoring purposes, respecting European Union and Italian regulations for animal welfare. Samples were not purposely collected for research experiments. Written consent was obtained by owners of the donkeys. Research protocols were approved by the University of Messina ethical committee for animal experimentation (n. 012014).

### Animals, samples and DNA extraction

Ion Proton and Ion Torrent PGM sequencing was carried out using DNA from 14 unrelated donkeys. One male Grigio Siciliano x Ragusano donkey (Peppe) and one male Grigio Siciliano x Ragusano x Ragusano donkey (Pippo) were used for whole genome sequencing using Ion Proton. Four Ragusano, four Grey (sampled in Sicily in the province of Messina in eight different farms) and four Martina Franca (sampled in Puglia in the province of Lecce from four different farms) donkeys were used for Ion Torrent sequencing of a RRL obtained from pooled DNA.

For all listed donkeys, whole blood sampled with EDTA as anticoagulant was stored at -80°C till DNA extraction. DNA extraction was done using the Wizard Genomic DNA Purification kit (Promega Corporation, Madison, WI, USA), following the manifacturer protocol for whole blood. DNA quantity and quality was assessed using a Nanophotometer P-330 (Implen GmbH, München, Germany) and the integrity of the extracted material was evaluated by visual inspection after agarose gel electrophosesis (0.8% in TBE1X) and gel red staining of 1.5 μg of DNA. Moreover, Illumina reads generated for Willy, a donkey reared in the Copenhagen zoo (Denmark), and used for a first draft donkey genome assembly [36] were also considered and analysed as described below.

### Ion Proton sequencing

Libraries for Peppe and Pippo were prepared following the manufacturer protocols for genome sequencing with the Ion Proton sequencer (Thermo Fisher Scientific/Life Technologies). Briefly 1.2 μg of genomic DNA was enzymatically sheared (8 min of incubation at 37°C), end repaired and adapter ligated using the Ion Xpress Plus Fragment Library kit (Thermo Fisher Scientific/Life Technologies). The resulting sheared and adapter ligated DNA material was then size selected using the e-gel system (Invitrogen, Carlsbad, CA, USA). In order to avoid bias due to the enzymatic shearing, two bands corresponding to 200 bp and 250 bp respectively (considering the insert size and the adapter) were collected for each donkey. Then, each size selected library was separately quantified by qPCR using a StepOnePlus Real-Time PCR System (Thermo Fisher Scientific/Life Technologies). Each size selected library was diluted according to the final concentration of 11 pM and clonally amplified using the Ion PI Template OT2 200 Kit v2 and Ion PI Template OT2 200 Kit v3 (Thermo Fisher Scientific/Life Technologies). Amplified libraries were then purified and sequenced on the Ion Proton sequencer for a total of five sequencing runs. For the first donkey (Peppe), two runs with the 200 bp library using Ion PI Chip (Thermo Fisher Scientific/Life Technologies) and one run with the 250 bp library using Ion PI Chip v2 (Thermo Fisher Scientific/Life Technologies) were done, for a total of 3 chips. For the second donkey (Pippo), one run with the 200 bp library (with the Ion PI Chip v2) and one run with the 250 bp library (with the Ion PI Chip v2) (Thermo Fisher Scientific/Life Technologies), for a total of 2 chips, using the Ion PI Sequencing 200 Kit v2 and v3 (Thermo Fisher Scientific/Life Technologies). Next generation sequencing data were deposited in the EMBL-EBI European Nucleotide Archive (ENA) with the project accession number PRJEB8743.

### Preparation of a Reduced Representation Library (RRL) and Ion Torrent sequencing

A DNA pool was prepared including equimolar DNA from 12 donkeys of three different breeds (4 Martina Franca, 4 Grey, 4 Ragusano donkeys). Then, aliquots of 1μg of this DNA pool were separately used for overnight digestions with several restriction enzymes and the digested products were loaded on 1% agarose gel, electrophoresed and visualized with 1X GelRed Nucleic Acid Gel Stain (Biotium Inc., Hayward, CA, USA). Two restriction enzymes (*Alu*I and *Rsa*I) did not produce visible patterns in the range of 200–400 bp that could be ascribed to repetitive elements (data not shown). DNA fragments from this range obtained from the indicated enzymes (purchased from Thermo Scientific/Fermentas, Vilnius, Lithuania) were purified from agarose gels with the QIA-quick Gel Extraction Kit (Qiagen, Hilden, Germany) according to the manufacturer’s instructions. Gel-recovered DNA was used for library preparation and sequencing. Briefly, two different libraries, one for each selected enzyme, were prepared following the instructions for Ion Torrent PGM (Thermo Fisher Scientific/Life Technologies) sequencing of short amplicons. For each library, 200 ng of amplified DNA was end-repaired and adapter-ligated with the Ion Plus Fragment Library kit (Thermo Fisher Scientific/Life Technologies). Then, each library was quantified by qPCR using a StepOnePlus Real-Time PCR System (Thermo Fisher Scientific/Life Technologies) with the Ion Library Quantitation Kit (Thermo Fisher Scientific/Life Technologies). The libraries were diluted to the final concentration of 26 pM, clonally amplified by emulsion PCR with the Ion One Touch 400 Template kit (Thermo Fisher Scientific/Life Technologies), purified and sequenced with the Ion PGM Sequencing 400 kit using a Ion 318 v2 chip (Thermo Fisher Scientific/Life Technologies), following the manufacturer protocols. Next generation sequencing data obtained from the RRL were deposited in the EMBL-EBI European Nucleotide Archive (ENA) with the project accession number PRJEB8744.

### Analysis of Ion Proton and Ion Torrent obtained reads and data from Willy

Sequencing reads obtained from the and Ion Torrent PGM runs were first automatically processed with the Ion Torrent Suite (TS) v4.1 on the Ion Torrent Server (Thermo Fisher Scientific/Life Technologies) including the following steps: i) polyclonal and low quality sequences were filtered; ii) adapters and low quality 3’-ends were trimmed from the high quality reads. Alignment of the trimmed reads with the horse reference genome (EquCab2, accession number GCA_000002305.1) was performed with the TMAP aligner [48] included in the TS v3.6 using the default options. Aligned files were created for each run using the EquCab2 (GCA_000002305.1) assembly and, for Peppe and Pippo, other aligned files were generated also using the draft donkey genome [36]. The *rmdup plugin* was then applied to eliminate duplicate reads from the aligned reads, in order to avoid redundant information. The animals individually sequenced were also aligned to the draft sequence of the donkey genome [36] and then the two different aligned output were processed in the same way for the following steps. Sequencing reads of Willy (retrieved from the Short Reads Archive, with the accession no. SRX290677, SRX290675, SRX290673 [36]) were aligned to the horse reference genome (EquCab2.0) and the draft donkey genome was done using BWA aln 0.7.4 and Samtools with the default options [49, 50].

Aligned donkey reads were used to identify sequence divergence with the EquCab2.0 horse genome version by counting differences. Sequence divergence was calculated in 1-Mbp windows on the EquCab2.0 genome version and plotted in a box plot representation for the different horse chromosomes. All data analysis and manipulation was carried out in the R environment [51] or using customized scripts.

### Single nucleotide polymorphism calling and statistical analyses

For each of the sequenced and aligned dataset described above the combination of the *mpileup* and *bcftools* functions of Samtools [50] was used for calling variants (SNPs) without any pre-set filter. Variants were called following these criteria: i) only nucleotide substitutions were considered, neglecting indels as Ion Torrent and Illumina sequencers poorly obtain concordant results for this variability [43]; ii) variation that obtained a mapping quality score ≥20 and a coverage ≥4X at the mutation point; iii), variation that were in homopolymeric regions, characterized by the presence of 4 or more contiguous identical nucleotides, were discarded. The latter filtering step was not applied to Illumina data because this technology is less prone to errors in short homopolymeric stretches [52]. All these analyses were done using Bash and Python scripts. Samtools options and Python scripts were also used to obtain information about Ion Proton reads aligned to the donkey Y chromosome scaffolds [36], avoiding potential homologous X chromosome regions. Single nucleotide polymorphism density was calculated in 1-Mbp windows across the EcuCab2.0 autosomes and chromosome X. A box plot representation was obtained as mentioned above. Analysis of every predicted variation was performed using the Variant Effect Predictor perl script and web interface [53] with the horse genome (EquCab2.0) as reference genome. To obtain a first evaluation of the SNP calling data 20 primer pairs were designed to target 20 randomly selected SNPs (included in putatively annotated donkey genome regions as deduced using information obtained from the EcuCab2.0 genome) and used to amplify and sequence amplicons obtained from a DNA pool obtained merging equimolar quantity of DNA extracted from Peppe and Pippo (S1 Table). Sequencing was carried out using the Sanger technology as previously described [45,46]. Obtained electropherograms were analysed using CodonCode Aligner (CodonCode Corporation, Dedham, MA, USA) and checked visually inspecting SNP positions.

## Results and Discussion

### Ion Proton sequencing of the genome of two donkeys and comparison with Illumina data

Two unrelated male donkeys (named Peppe and Pippo) reared in Sicily that were hybrids of two Sicilian donkey populations (a Grigio Siciliano x Ragusano and a Grigio Siciliano x Ragusano x Ragusano) were used for semiconductor-based sequencing of their genomes. Hybrids of two breeds (Grigio Siciliano has a low number of pure animals and Ragusano is probably the largest Italian population) were selected to increase the possibility to identify polymorphisms (see below), as pure breeds might be quite inbred [21]. In addition, hybrid populations or not very well genetically defined donkeys are very common among Italian donkey populations as also observed in many other countries [6]. The total number of reads (and related metrics) obtained from the generated libraries and Ion PI Chip (Thermo Fisher Scientific/Life Technologies) runs for the two sequenced donkeys is reported in S2 Table.

**Fig 1:**
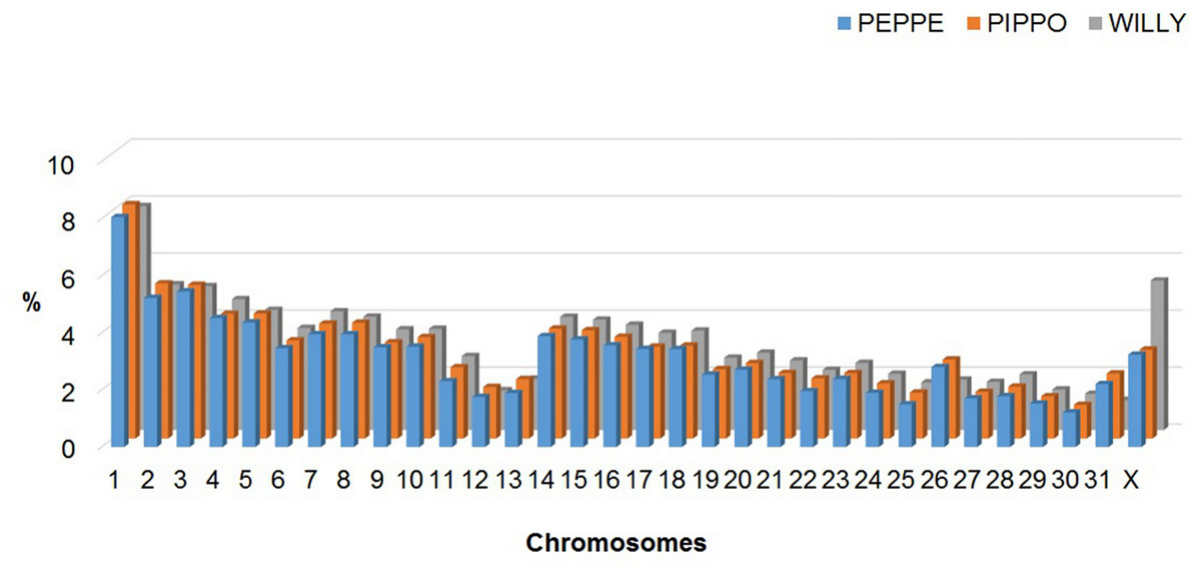
Distribution (%) of the mapped donkey reads on the EquCab2.0 chromosomes. Reads for two donkeys (Peppe and Pippo) were produced with the Ion Proton sequencer and compared with mapped reads obtained from Willy and produced with the Illumina instrument as reported by [36]).

**Fig 2:**
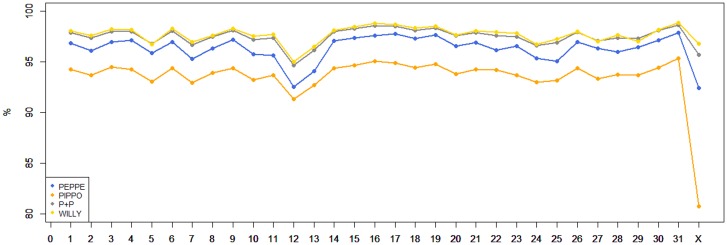
Coverage of the mapped donkey reads on the EquCab2.0 chromosomes. Data have been reported for the different chromosomes and for the three sequenced donkeys (Peppe and Pippo with Ion Proton and Willy with Illumina [36]). P+P (the merged sequences output of Peppe and Pippo) is almost complitery overlapping the resuts of the output of Willy, therefore it is not always directly visible in the figure.

The first draft assembly of the donkey genome reported in Orlando et al. [36] and generated from Illumina sequences of a donkey (Willy) reared in the Copenhagen zoo (Denmark) is based on 90,287 scaffolds and 420,214 shorter contigs. All the scaffolds account for a total of about 2.29 Gbp while the contigs account for 60.48 million of nucleotides for an overall total of 2.35 Gbp. The N50 size of the contigs and of the scaffold is 6.38 kb and 100.94 kb respectively. The EquCab2.0 horse genome has 2.37 Gbp and its N50 size of the contigs (no. = 55,316) and of the scaffolds (no. = 9,687) is 112.38 kbp and 46 Mbp, respectively [27]. These features affected the number of reads we obtained from the Ion Proton runs of the two donkeys that were aligned to the two *Equus* genomes (Table 1). Considering the two animals together, a total of about 214.15 million of reads were aligned to the draft donkey genome (scaffolds+contigs) whereas about 8.48 million of reads were not mapped, using the stringent procedure adopted (see Methods). The same analysis against the horse reference genome (EquCab2.0, including the autosomes and the chromosome X), produced 236.67 million of Ion Proton mapped reads and only 2,286 unmapped reads. Mean depth of the produced reads was 11.06 X with 94.76% coverage and 12.22 X with 97.54% coverage considering the draft donkey and the EquCab2.0 genome versions, respectively. A lower number of Illumina donkey reads aligned to the draft donkey scaffolds than those aligned to the EquCab2.0 genome was already reported by Orlando *et al*. [36], despite the draft genome of *E*. *asinus* was produced from the same Illumina reads used for the *de novo* assembly of this genome. This is probably due to the very preliminary assembly attempted for the donkey genome, that, however, was useful to deduce evolutionary information [36]. For a comparison, we aligned these Illumina reads obtained for the donkey genome [36] against the EquCab2.0 genome and obtained 538,783,052 mapped reads (with a mean depth coverage of 15.85 X). A summary of the mapped donkey Ion Proton and Illumina reads on the horse autosomal and X chromosomes available in the EcuCab2.0 genome version and their chromosome coverage are reported in Figs 1 and 2, respectively. Details are reported in S3 Table.

**Table 1:** Ion Proton reads obtained from the genome of the two sequenced donkeys.

**Aligment with the draft donkey genome [36]**				
	No. of reads (unaligned)	Nucleotides	Mean depth (X)	Coverage (%)
Peppe	144,087,558 (6,496,365)	17,073,700,000	7.27	93.53
Pippo	70,058,654 (1,984,507)	8,898,950.,000	3.79	90.21
Peppe+Pippo	214,146,212 (8,480,872)	25,972,650,000	11.06	94.76
**Aligment with the EcuCab2.0 horse genome**				
	No. of reads (unaligned)	Nucleotides	Mean depth (X)	Coverage (%)
Peppe	159,629,721 (1,355)	19,012,008,351	8.03	96.33
Pippo	77,041,062 (931)	9,919,701,614	4.19	93.29
Peppe+Pippo	236,670,783 (2,286)	28,931,709,965	12.22	97.54

Reads are aligned with the draft donkey and EquCab2.0 horse genomes and related metrics are reported.

### Genome wide nucleotide divergence between the horse and donkey genomes

**Fig 3:**
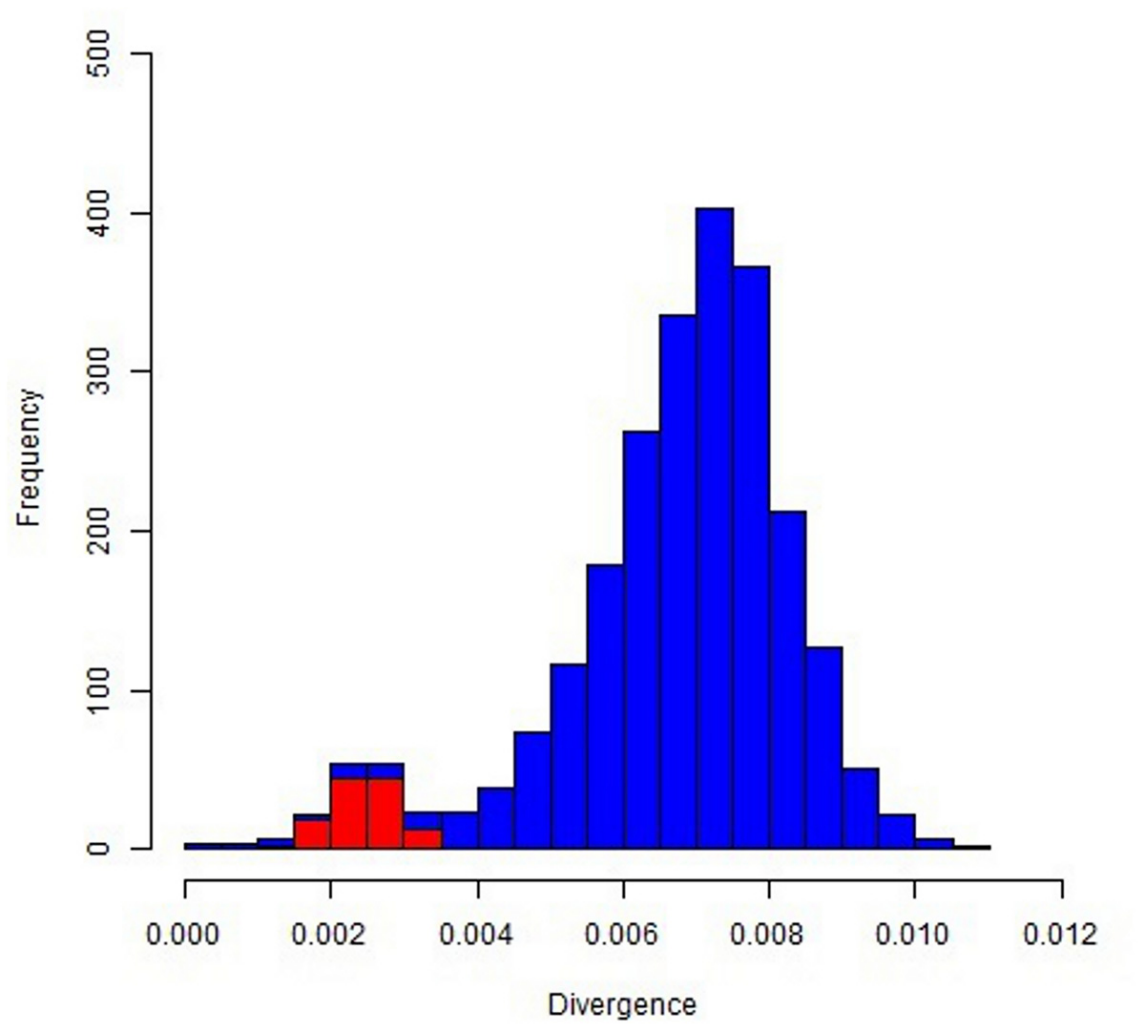
Nucleotide divergence between horse and donkey genomes: distribution of divergence rates in 1-Mb chromosome regions designed on the EcuCab2.0 genome version. Autosomal windows are in blue and chromosome X windows are in red.

**Fig 4:**
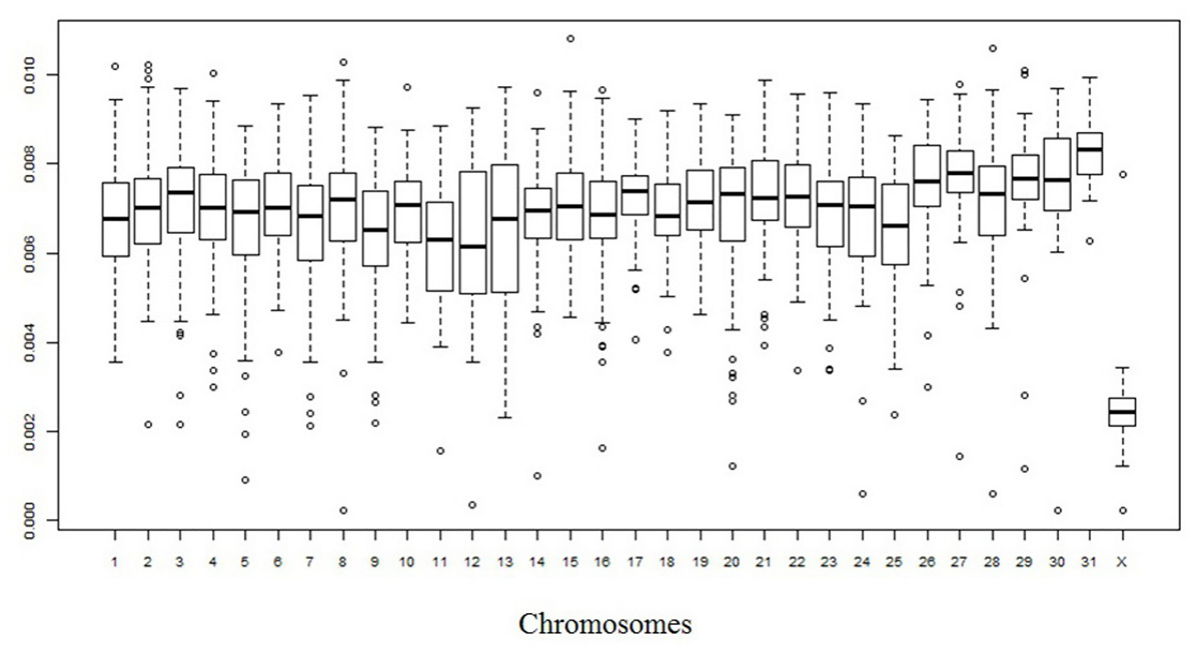
Nucleotide divergence between horse and donkey genomes: box plot of the distribution by chromosomes (x axis) of the divergence rate in 1-Mb windows (y axis). Quartiles are the edges of the box. The edges of the boxes correspond to quartiles; the notches are the standard errors of the median; and the vertical bars to the range.

Considering only fixed single nucleotide differences between the EquCab2.0 genome and the obtained Ion Proton donkey sequences, a total of about 18.28 million positions were different between the two species. If we use only Illumina reads obtained from Willy [36], the number of differences with the EquCab2.0 genome was about 22.12 M, similar to what obtained with the Ion Proton. We were a little bit more stringent than Orlando *et al*. [36] who reported about 23.8 M of different positions. S3 Table also reports the detailed information separated for the different horse chromosomes. S1 Fig shows the averaged density of differences between the horse and donkey genomes across chromosomes whereas Figs 3 and 4 show the distribution of divergence rates in 1-Mb chromosome regions designed on the EcuCab2.0 genome version. If we combine the three sequenced donkey genomes (two that we sequenced with Ion Proton and one obtained with Illumina [36]), the number of fixed positions were reduced to a total of about 15.9 million, that means that genome wide nucleotide divergence *α* between *E*. *caballus* and *E*. *asinus* could be ~0.67%. However, it could be possible that a fraction of the fixed differences might be polymorphic in donkey as well as in horse, reducing this estimation of about 0.10–0.15%, depending on the effective population size of the two species (reaching a level of ~ 0.52–0.57% of nucleotide divergence between these species; see also below the identification of SNPs). This number is lower than that reported between the human and chimpanzee genomes and obtained from a more extensive analysis (1.06%) [54], two species that diverged about 6–7 Myr BP [54, 55]. The genome wide nucleotide divergence between horse and donkey, as roughly estimated in our study, reflects the more recent differentiation of these two species (4.0–4.5 Myr BP [36]) than what estimated as time of divergence between the human and the chimpanzee lineages. Orlando *et al*. [36] also suggested slower mutation rates in horse than human. Based on this, and comparing the human-chimpanzee nucleotide divergence, our estimation of about 0.52%-0.57% of differences between horse and donkey seems to fit well the *Equus* most recent common ancestor living about 4.0–4.5 Myr BP [36].

Variation in nucleotide divergence rate between donkey and horse, as we approximated using detected fixed single nucleotide differences, could be present among different chromosomes and chromosome regions (Figs 3 and 4, S1 Fig, S1 and S4 Tables). The average divergence in 1-Mb windows (determined on the horse chromosomes) ranged from 0.021% to 1.081% with a standard deviation of 0.165%, that is larger than what would be expected assuming a uniform divergence rate.

The striking value observed for chromosome X (a mean divergence of about 0.25%, less than half the divergence estimated for the autosomal chromosomes) can be explained by the lower mutation rate of chromosome X [56]. A 2:1 ratio between autosomes and chromosome X was already reported for the nucleotide divergence between human and chimpanzee [54]. Anyway, the quite lower value for the horse *vs* donkey chromosome X (~2.5:1 ratio between autosomes and chromosome X) might be derived by the lower effective population size for these two species and/or could be due to frequent chromosome X flow during the speciation events [57]. This latter hypothesis could be in part supported by a similar distribution of nucleotide divergence classes between autosomal windows and the chromosome X windows, producing a bimodal-like distribution for the autosomal windows (Figs 3 and 4). Further studies are needed to better clarify this distribution and to evaluate if these potential signatures of evolutionary constrains might have a biological relevance according to the genes included in these regions. A higher nucleotide divergence rate between donkey and horse would be expected for chromosome Y due to the higher mutation rate in the male than in the female germ line [54, 55]. However, as chromosome Y is not included in the EquCab2.0 horse genome version and only partial Y-chromosome sequences are available for the horse from other studies (i.e. [58, 59]), a comprehensive analysis of differences between *E*. *asinus* and *E*. *caballus* for this chromosome was not attempted (see also below for further evaluations of Y-chromosome sequences). Non-constant nucleotide divergence rates across the autosomal genome was also evident (Figs 3 and 4). This could be due to differences in i) CpG content, ii) G + C content, iii) gene densities and iv) recombination rates in different chromosome or chromosome regions and/or large scale-chromosome structures as also observed in other species [54, 55]. Other studies should be carried out to evaluate in more details these questions but a more precise assembly of the donkey genome would be needed for a more detailed analysis of the evolutionary aspects that might have contributed to shape this species.

### Y-chromosome sequence information

As the two Ion Proton sequenced donkeys and the Illumina sequenced donkey were males, we analyzed Y-chromosome sequences to identify sequencing errors that could be useful to estimate the error rate of the Ion Proton sequencing platform and to identify SNPs that might be useful to track paternal lineages in the donkey. The first aim was reached comparing within animals Y-chromosome sequences whereas the second aim was reached comparing between animals Y-chromosome sequences.

The draft donkey genome reported by Orlando *et al*. [36] identified a total of 703 Y-chromosome scaffolds (~5.87 Mbp), 347 of which did not match any scaffolds assigned also to chromosome X (3,033,416 bp). A total of 2,300,362 reads obtained from Peppe + Pippo were mapped on chromosome Y. Only 217,547 reads mapped to Y-specific scaffolds only, for a mean depth of 7.17 X for Peppe and 3.23 X for Pippo (coverage was 87.94% and 72.98%, respectively). In this way, we were able to reduce the potential problems derived by partial homology (or by mapping problems) between the two sex chromosomes that might produce biases in the subsequent evaluations.

Y-chromosome sequences that might be hemizygous in the sequenced males (only one copy per genome) were used to identify sequencing errors looking at heterozygote positions (that should not be present) in the separate analyses of the data from the two Ion Proton sequenced donkeys. Based on this comparison, the estimated error rate was 0.063%. However, it is worth mentioning that our filtering that wanted to extract Y-specific chromosomes (considering the limits derived by the poorly characterized and distinguished sex chromosome sequences in donkey that mainly relied on horse sequence information) might have attributed erroneously reads to Y chromosome regions, overestimating this error rate.

To identify differences among Y-chromosomes from the different animals, considering only regions with depth ≥ 3, a total of 1,362 and 798 single nucleotide variants (SNVs) were identified in the Y-specific regions comparing the draft donkey genome with sequences obtained for Peppe and Pippo, respectively. Of these differences, 311 SVNs were in common between the two animals. These data indicated that different paternal lineages distinguished the three considered donkeys (Peppe, Pippo and Willy [36]). These Y-chromosome SNVs could be important to better define the domestication processes that differentiated wild asses from domestic donkeys. Other study will be needed to better define Y-chromosome population structures in different donkey breeds and populations.

### Identification of single nucleotide polymorphisms in donkeys

Using a stringent approach that excluded indels and homopolymeric regions and considered a coverage of at least ≥ 4X (see Materials and methods; see also [60]), whole genome resequencing data (excluding Y chromosome sequences) from the two Ion Proton sequenced donkeys produced a total of ~4.1 million of autosomal SNPs (S5 Table). The Illumina data from Willy reported ~2.2 million of SNPs (S5 Table). Using the same Illumina dataset, Orlando *et al*. [36] reported ~2.3 million of SNPs, that means that we were a little bit more stringent in calling polymorphisms. Intersection between data obtained for the three donkeys is reported in the Venn diagram of S2 Fig. To evaluate the quality of these results, the transition/transversion (Ti/Tv) ratio was calculated. With the Ion Proton filtered genome wide data the Ti/Tv ratio was 2.26, a value that is comparable to what we obtained in another genome sequencing study carried out in pigs using the same sequencing technology (2.08; [46]). These values are similar to what DePristo *et al*. [60] reported for the human genome (Ti/Tv ratios in whole genome sequencing of 2.07 and 2.1 for novel or already identified SNPs, respectively) and are comparable to the ratios obtained by several other next generation sequencing studies produced with the Illumina technology for which transitions were about twice more frequent than transversions (i.e. [61, 62]). Using the donkey Illumina reads obtained from Willy, we calculated the Ti/Tv = 1.95, confirming the ~2:1 ratio obtained with the Ion Proton reads. A few differences between these parameters determined with the two sequencing platforms (Ion Proton and Illumina) might be derived by different sequencing biases as previously reported by others who, anyway, reported 96% concordance in SNP detection [43].

To obtain additional information on SNPs in the donkey genome and to indirectly validate a portion of SNPs identified by whole genome resequencing, two RRLs were produced using 12 donkeys of three different breeds (Ragusano, Grigio Siciliano and Martina Franca, another Italian population originated in the Puglia region) and sequenced with the Ion Torrent PGM. A total of ~8.22 million reads having base quality ≥ 20 (~1.2 Gbp) were aligned to the EcuCab2.0 horse genome. About 0.55 Gbp were aligned with at least ≥ 4 X depth. A total of 41,276 heterozygous SNPs (38,894 on autosomes) were identified among the donkey reads obtained from the two RRLs (S5 Table). About 43% of these autosomal SNPs (16,833 out of 38,894) were also detected by whole genome resequencing obtained by the Ion Proton sequencer. The number of the RRL autosomal SNPs detected also in Willy was 5,690 (about 15%). The total number of SNPs that were detected considering the three animals was 18,860. This means that the RRL strategy was able to capture, in proportion, about 50% of additional SNPs, giving an approximate indication of the level of variability that might be present in the donkey genome. To further validate the identification of SNPs derived by the next generation semiconductor sequencing reads, 20 randomly selected polymorphisms belonging to coding sequences of several genes were also analysed by Sanger sequencing (S1 Table). The results confirmed the reliability of the filtering and calling procedures for SNP detection as all Sanger sequenced SNPs were confirmed.

**Fig 5:**
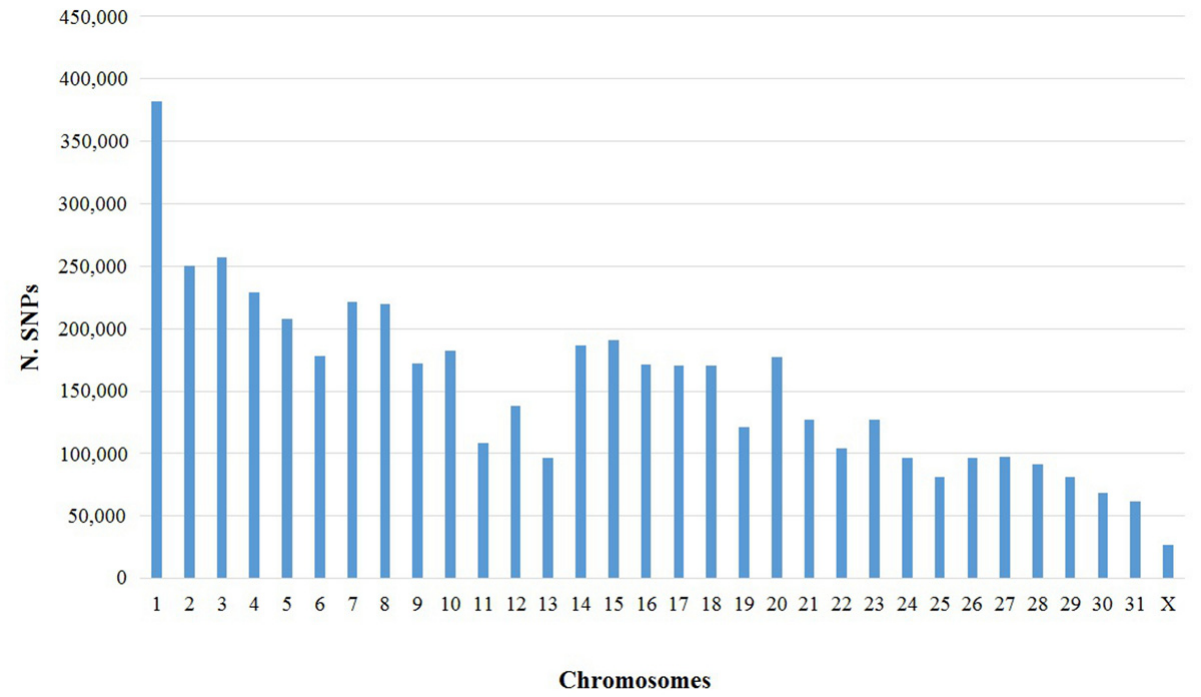
Distribution of donkey single nucleotide polymorphisms (SNPs). Distribution of SNPs is based on the different horse chromosomes (EquCab2.0 genome version).

**Fig 6:**
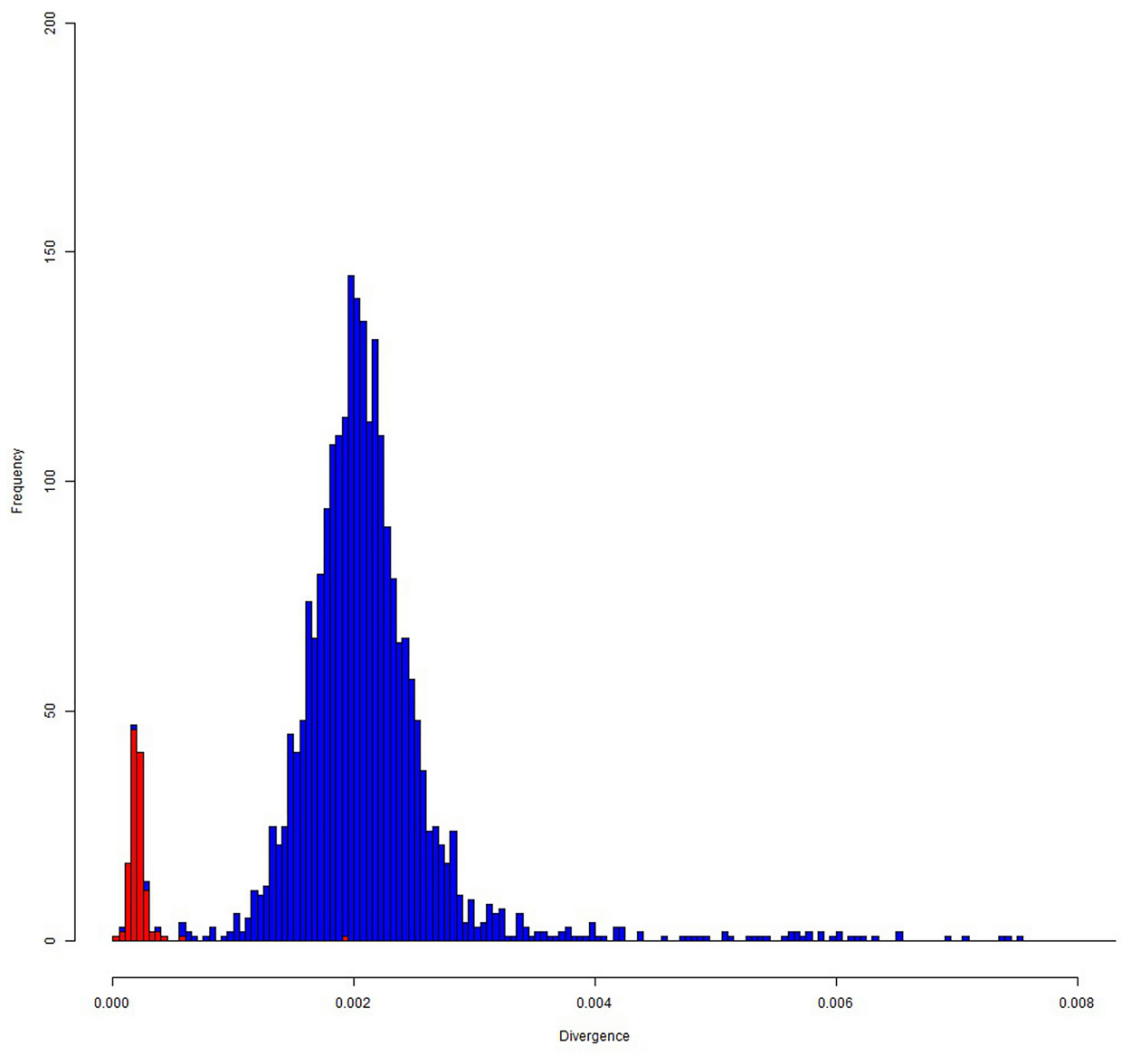
Densities of donkey single nucleotide polymorphisms: Distribution of the donkey SNP density in 1-Mb chromosome windows, across the EquCab2.0 genome. Autosomal windows are in blue and chromosome X windows are in red.

**Fig 7:**
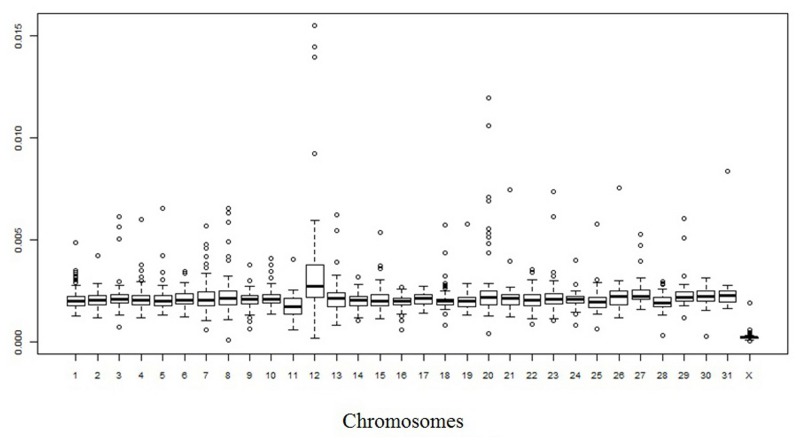
Densities of donkey single nucleotide polymorphisms: Box plot of the distribution by chromosomes of the SNP density in 1-Mb windows. Quartiles are the edges of the box. The edges of the boxes correspond to quartiles; the notches are the standard errors of the median; and the vertical bars to the range.

The total number of SNPs divided by horse chromosomes and their densities are reported in Figs 5, 6 and 7, respectively. A comparison between autosomal and X chromosome SNPs detected in the donkey genome with differences between the horse and donkey genome, projected on the horse chromosomes can be obtained from Fig 7 and S1 Fig. It is interesting to note that for the corresponding donkey reads mapping on horse chromosome 12 (ECA12), a higher density of SNPs was observed in all three donkeys and two sequencing platforms, reducing the possibilities of a systematic error. The reason for this higher SNP density is not known. However, it is interesting to note that four independent studies [63–66] reported that ECA12 was the most enriched horse chromosome of copy number variations (CNVs). Copy number variation might contribute to increase the level of variability in different allelic copies that can subsequently evolve in the constitution of gene duplications [67]. The ECA12 is homologous with the donkey chromosome 17 (EAS17), as demonstrated by comparative FISH mapping and chromosome specific painting experiments between these two species [30–32]. Considering that in close species conserved structural duplications could produce recurrent interspecies CNV [68, 69], a high density of CNV might be also present in EAS17, producing also a higher density of single nucleotide variants that cannot be distinguished from true allelic SNPs using only sequencing reads. Distribution of different SNP densities across chromosomes and chromosome regions are evident from Figs 6 and 7. The level of SNP density seems to follow in parallel the nucleotide divergence ratio between horse and donkeys. In particular a lower SNP density can be observed on chromosome X, as expected according to previous studies in other mammals [54, 56, 70].

**Fig 8:**
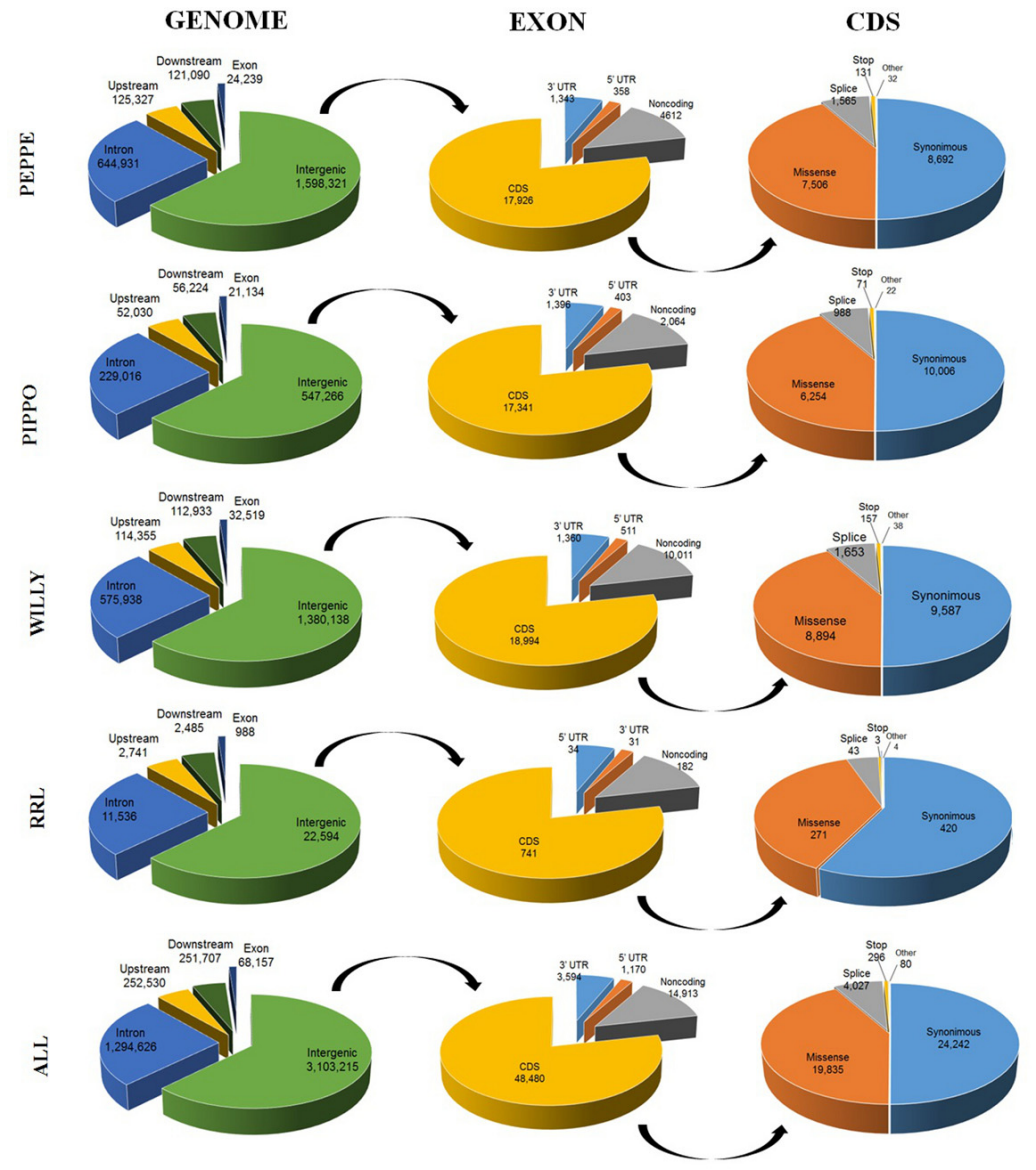
Annotation of the identified single nucleotide polymorphisms (SNPs) and their distribution according to the different putative function or position. Data are reported for the two sequenced donkeys with Ion Proton (Peppo and Pippo), Willy (for which SNPs data were annotated in this study, using Illumina reads obtained by Orlando *et al*. [36]), the reduced representation library (RRL) data and all SNPs together.

Annotation of the identified SNPs was obtained using the Variant Effect Predictor (VEP) [53]. A total of ~4.8 million of donkey SNPs were evaluated with this tool that reported more than 5.0 million of predicted effects (some positions were associated with more than one predicted effect). The largest number of variants were intergenic (3.1 million) or intronic (1.3 million). In coding regions, a total of 23,923 synonymous and 19,536 missense variants were predicted in a total of 5,699 different annotated genes (as deduced from the horse genome annotation). Fig 8 summarizes the VEP results for the two Ion Proton sequenced donkeys (Peppo and Pippo), for Willy (obtained by our analysis of the Illumina reads retrieved from [36]), for the RRL data and for all information gathered together. As examples of VEP annotated SNPs, S6 Table lists all autosomal missense, splice and stop gain/loss mutations identified by merging all datasets. Many of these mutations might have a functional relevance. It will be interesting to evaluate the distribution of these relevant mutations in different donkey populations.

## Conclusions

In this study we used next generation sequencing data generated from the Ion Proton and Ion Torrent sequencers to describe, at the whole genome level, SNPs in the *E*. *asinus* genome. This study represents one of the first in which the Ion Proton sequencer was used to investigate a complex and large genome. Data we produced from these next generation semiconductor sequencing platform were combined with Illumina whole genome reads that were previously used to construct a first draft of the donkey genome [36]. The percentage of nucleotide divergence between horse and donkey was estimated using sequencing data. Regions with low nucleotide divergence were identified in several autosomal chromosomes in addition to the whole chromosome X. These regions might be evolutionally important in equids. Additional studies are needed to understand the biological reasons of this low level of divergence between these species in some parts of the equid genomes. Moreover, a few millions of SNPs were identified and annotated. SNP densities and nucleotide divergence ratios distributed across the donkey chromosomes were similar. These polymorphisms will constitute a first resource useful to describe variability at the population genomic level in this species and to establish monitoring systems for the conservation of donkey genetic resources, integrating microsatellite variability data. According to what we reported and described, the refinement and improvement of the assembly of the donkey genome, including an extensive annotation, would positively affect subsequent studies investigating the donkey genome.

## Supporting Information

S1 Fig Nucleotide divergence between horse and donkey and densities of donkey single nucleotide polymorphisms (SNPs). Averaged chromosome-wide nucleotide horse-donkey divergence rates and donkey SNP densities across chromosomes. SNP densities are plotted for the three sequenced donkeys (Peppe and Pippo with Ion Proton and Willy with Illumina [36]).
(TIFF)

S2 Fig Venn diagram showing the intersection among the identified single nucleotide polymorphisms in the sequenced donkeys. (TIFF)

S1 Table Donkey genome regions sequenced by Sanger sequencing to validate identified single nucleotide polymorphisms. Primers used for Sanger sequencing and coordinates of the sequenced regions reported in the corresponding positions of the EquCab2.0 horse genome version.
(XLSX)

S2 Table Sequenced reads and nucleotides obtained from the Ion Proton runs. Summary of the produced reads and nucleotides after adapter trimming and filtering from the TS v4.1.
(DOCX)

S3 Table Metrix and statistics of the sequenced donkeys. Number of reads, mean coverage and depth considering the EquCab2.0 horse reference genome (divided by chromosomes) for the two Ion Proton sequenced donkeys (Peppe and Pippo, including merged data, P&P) and for the donkey sequenced with Illumina by Orlando *et al*. [36].
(XLSX)

S4 Table Number of fixed differences between the donkey sequenced genomes and the EquCab2.0 horse genome version. Information is reported for the two merged Ion Proton sequenced donkeys (Peppe and Pippo), for the Illumina sequenced donkey (Willy [36]) and for the combination of the three sequenced donkeys (combined), divided by horse chromosomes (ECA) as reported in EquCab2.0. Fixed differences were considered those that were homozygous in the sequenced donkey genomes compared to the EquCab2.0 horse genome version.
(DOCX)

S5 Table Number of single nucleotide polymorphisms identified in the Ion Proton and Illumina sequenced donkeys. Information divided by the corresponding autosomal horse chromosomes (ECA) is reported separately or merged for the two Ion Proton sequenced donkeys (Peppe and Pippo), for the Illumina sequenced donkey (Willy [36]) and for the Reduced Representation Library (RRL).
(DOCX)

S6 Table Results of the Variant Effect Predictor (VEP) analysis for all single nucleotide polymorphisms (SNPs) identified in coding regions. SNP data were derived by the combination of the three sequenced donkeys (Peppe, Pippo and Willy) and including also reduced representation library results. Annotation was obtained from EquCab2.0.
(XLSX)
