# Supplementary figures and images for: Next Generation Semiconductor Based Sequencing of the Donkey (Equus asinus) Genome Provided Comparative Sequence Data against the Horse Genome and a Few Millions of Single Nucleotide Polymorphisms
Source: PLoS One. 2015 Jul 7;10(7):e0131925. doi: 10.1371/journal.pone.0131925 (PMC4495037; doi:10.1371/journal.pone.0131925)

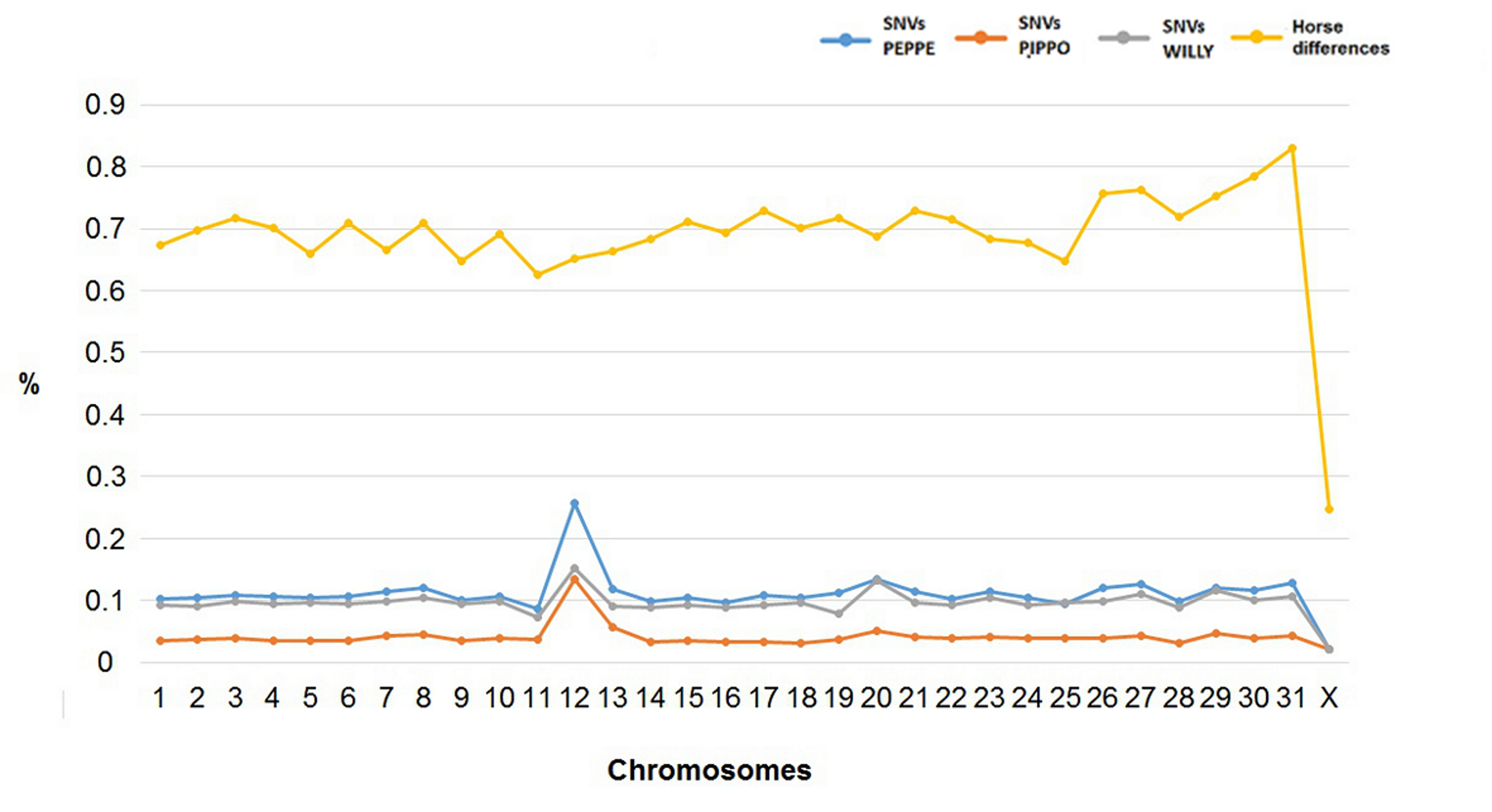

Supplement: S1 Fig — Averaged chromosome-wide nucleotide horse-donkey divergence rates and donkey SNP densities across chromosomes. SNP densities are plotted for the three sequenced donkeys (Peppe and Pippo with Ion Proton and Willy with Illumina [36]). (TIFF) [file pone.0131925.s001.tiff]

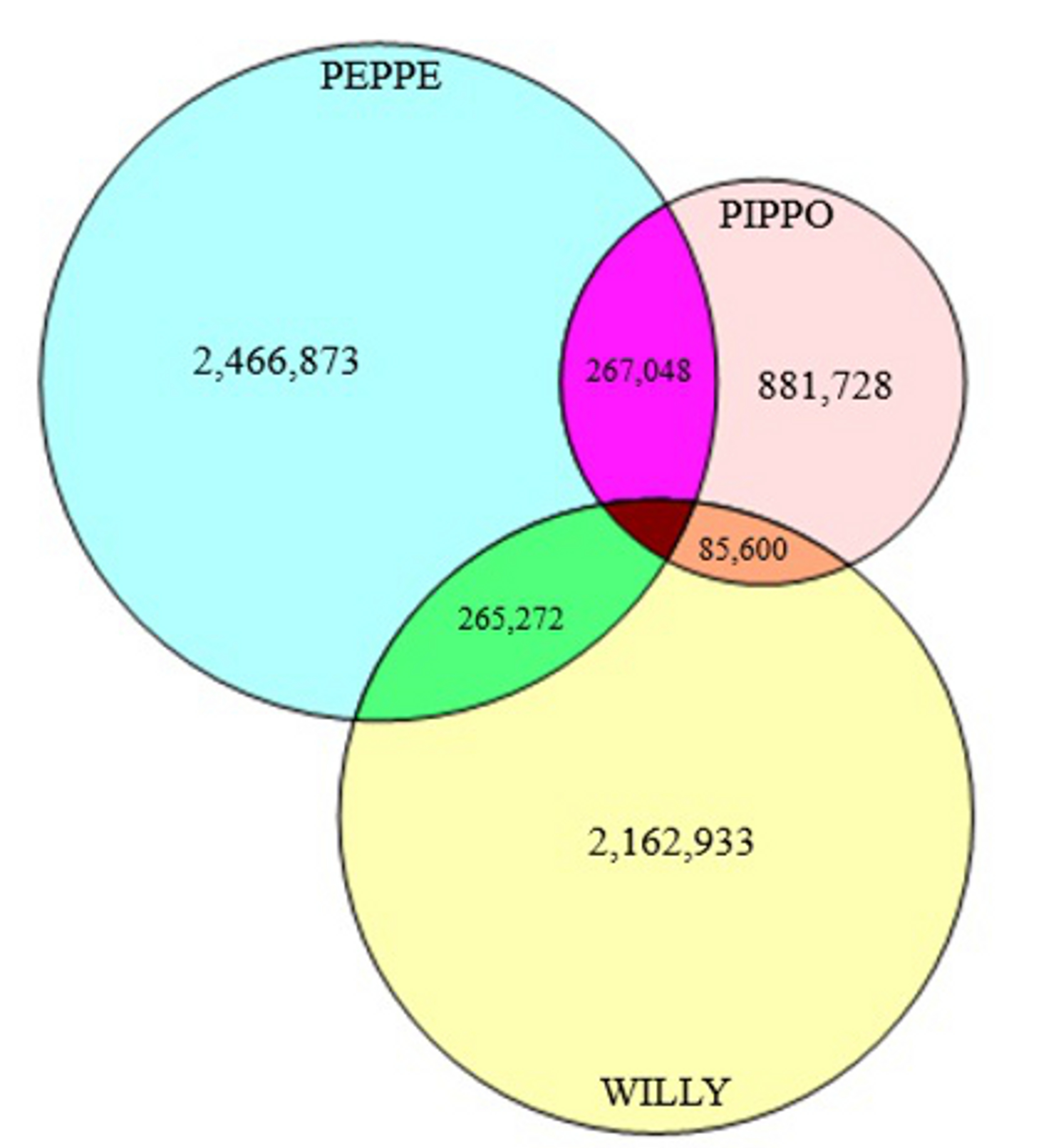

Supplement: S2 Fig — (TIFF) [file pone.0131925.s002.tiff]
