# Supplementary material for: Next Generation Semiconductor Based Sequencing of the Donkey (Equus asinus) Genome Provided Comparative Sequence Data against the Horse Genome and a Few Millions of Single Nucleotide Polymorphisms
Source: PLoS One. 2015 Jul 7;10(7):e0131925. doi: 10.1371/journal.pone.0131925 (PMC4495037; doi:10.1371/journal.pone.0131925)
Supplement: S2 Table — Summary of the produced reads and nucleotides after adapter trimming and filtering from the TS v4.1. (DOCX) [file pone.0131925.s004.docx]

**S2 Table. Sequenced reads and nucleotides obtained from the Proton Torrent runs.**

Summary of the produced reads and nucleotides after adapter trimming and filtering from the TS v4.1.

| **Donkey** | **Ion PI**  **chip runs** | **Total no. of reads (millions)** | **Mean read length (bp)** | **Total sequenced nucleotides (Gbp)** | **Total sequenced Q20 nucleotides (Gbp)** |
| --- | --- | --- | --- | --- | --- |
| Peppe | 1 | 77.2 | 127 | 9.78 | 7.72 |
|  | 2 | 67.4 | 125 | 8.42 | 6.69 |
|  | 3 | 67.6 | 121 | 8.18 | 6.39 |
| Pippo | 1 | 43.5 | 152 | 6.59 | 5.76 |
|  | 2 | 58.4 | 121 | 7.09 | 6.25 |
