# Supplementary material for: Next Generation Semiconductor Based Sequencing of the Donkey (Equus asinus) Genome Provided Comparative Sequence Data against the Horse Genome and a Few Millions of Single Nucleotide Polymorphisms
Source: PLoS One. 2015 Jul 7;10(7):e0131925. doi: 10.1371/journal.pone.0131925 (PMC4495037; doi:10.1371/journal.pone.0131925)
Supplement: S4 Table — Information is reported for the two merged Ion Proton sequenced donkeys (Peppe and Pippo), for the Illumina sequenced donkey (Willy [36]) and for the combination of the three sequenced donkeys (combined), divided by horse chromosomes (ECA) as reported in EquCab2.0. Fixed differences were considered those that were homozygous in the sequenced donkey genomes compared to the EquCab2.0 horse genome version. (DOCX) [file pone.0131925.s006.docx]

**S4 Table. Number of fixed differences between the donkey sequenced genomes and the EquCab2.0 horse genome version.**

Information is reported for the two merged Proton Torrent sequenced donkeys (Peppe and Pippo), for the Illumina sequenced donkey (Willy [36]) and for the combination of the three sequenced donkeys (combined), divided by horse chromosomes (ECA) as reported in EquCab2.0. Fixed differences were considered those that were homozygous in the sequenced donkey genomes compared to the EquCab2.0 horse genome version.

| **ECA** | **Proton Torrent** | **Willy** | **Combined** |
| --- | --- | --- | --- |
| 1 | 1,409,578 | 1,723,592 | 1,252,998 |
| 2 | 967,792 | 1,157,196 | 842,226 |
| 3 | 974,230 | 1,166,015 | 857,624 |
| 4 | 847,896 | 1,042,659 | 761,199 |
| 5 | 738,957 | 899,537 | 656,455 |
| 6 | 677,340 | 821,954 | 600,263 |
| 7 | 755,473 | 916,675 | 656,452 |
| 8 | 767,252 | 920,239 | 667,389 |
| 9 | 598,634 | 816,272 | 540,482 |
| 10 | 664,919 | 664,919 | 581,022 |
| 11 | 452,589 | 534,696 | 383,091 |
| 12 | 267,214 | 313,296 | 215,494 |
| 13 | 346,384 | 397,174 | 282,489 |
| 14 | 719,388 | 873,017 | 640,696 |
| 15 | 728,772 | 891,776 | 651,243 |
| 16 | 674,067 | 819,007 | 605,171 |
| 17 | 653,733 | 798,812 | 588,051 |
| 18 | 642,737 | 790,876 | 578,441 |
| 19 | 473,849 | 586,400 | 430,055 |
| 20 | 500,052 | 616,900 | 440,776 |
| 21 | 473,386 | 572,879 | 420,134 |
| 22 | 415,209 | 490,907 | 356,838 |
| 23 | 425,304 | 528,154 | 381,213 |
| 24 | 365,094 | 433,396 | 316,325 |
| 25 | 306,870 | 358,426 | 256,367 |
| 26 | 359,768 | 441,610 | 316,767 |
| 27 | 338,982 | 416,083 | 304,547 |
| 28 | 385,490 | 454,160 | 332,304 |
| 29 | 283,228 | 343,900 | 253,414 |
| 30 | 267,923 | 322,225 | 235,794 |
| 31 | 231,133 | 280,272 | 207,356 |
| X | 496,228 | 582,927 | 307,641 |
