# Supplementary material for: Next Generation Semiconductor Based Sequencing of the Donkey (Equus asinus) Genome Provided Comparative Sequence Data against the Horse Genome and a Few Millions of Single Nucleotide Polymorphisms
Source: PLoS One. 2015 Jul 7;10(7):e0131925. doi: 10.1371/journal.pone.0131925 (PMC4495037; doi:10.1371/journal.pone.0131925)
Supplement: S5 Table — Information divided by the corresponding autosomal horse chromosomes (ECA) is reported separately or merged for the two Ion Proton sequenced donkeys (Peppe and Pippo), for the Illumina sequenced donkey (Willy [36]) and for the Reduced Representation Library (RRL). (DOCX) [file pone.0131925.s007.docx]

**S5 Table. Number of single nucleotide polymorphisms identified in the Proton Torrent and Illumina sequenced donkeys.**

Information divided by the corresponding autosomal horse chromosomes (ECA) is reported separately or merged for the two Proton Torrent sequenced donkeys (Peppe and Pippo), for the Illumina sequenced donkey (Willy [36]) and for the Reduced Representation Library (RRL).

| **ECA** | **Peppe** | **Pippo** | **Peppe + Pippo** | **Willy** | **RRL** |
| --- | --- | --- | --- | --- | --- |
| 1 | 190,328 | 66,120 | 322,430 | 172,022 | 2,987 |
| 2 | 126,800 | 44,630 | 215,462 | 108,361 | 1,856 |
| 3 | 129,477 | 45,495 | 214,392 | 117,284 | 2,319 |
| 4 | 116,201 | 37,814 | 187,842 | 103,039 | 1,629 |
| 5 | 103,483 | 35,124 | 172,184 | 96,338 | 1,522 |
| 6 | 90,238 | 30,556 | 150,350 | 79,474 | 1,297 |
| 7 | 112,800 | 43,045 | 189,332 | 96,249 | 1,743 |
| 8 | 112,862 | 41,547 | 184,401 | 98,667 | 1,753 |
| 9 | 84,257 | 28,839 | 142,032 | 79,758 | 1,305 |
| 10 | 89,899 | 32,411 | 152,512 | 82,127 | 1,417 |
| 11 | 53,562 | 22,570 | 97,936 | 44,270 | 1,279 |
| 12 | 85,275 | 44,527 | 121,770 | 50,589 | 1,611 |
| 13 | 50,579 | 24,108 | 86,760 | 38,329 | 1,483 |
| 14 | 92,472 | 31,535 | 156,275 | 83,792 | 1,224 |
| 15 | 94,982 | 32,303 | 159,681 | 85,456 | 1,438 |
| 16 | 84,402 | 28,910 | 144,369 | 77,629 | 1,228 |
| 17 | 87,552 | 26,390 | 143,936 | 75,258 | 1,024 |
| 18 | 85,980 | 24,895 | 140,729 | 79,586 | 1,257 |
| 19 | 67,538 | 22,602 | 106,508 | 47,270 | 1,098 |
| 20 | 86,484 | 32,856 | 143,769 | 84,453 | 1,222 |
| 21 | 65,970 | 23,549 | 108,775 | 55,487 | 999 |
| 22 | 51,248 | 19,717 | 90,473 | 45,919 | 881 |
| 23 | 63,814 | 22,577 | 103,191 | 58,684 | 913 |
| 24 | 48,451 | 18,531 | 78,624 | 43,006 | 894 |
| 25 | 37,520 | 15,493 | 67,571 | 37,985 | 698 |
| 26 | 50,488 | 16,163 | 81,070 | 40,855 | 884 |
| 27 | 50,745 | 16,866 | 81,422 | 44,481 | 510 |
| 28 | 45,880 | 14,307 | 78,770 | 40,730 | 642 |
| 29 | 40,695 | 16,135 | 66,007 | 39,451 | 813 |
| 30 | 34,780 | 11,542 | 58,420 | 29,971 | 420 |
| 31 | 32,111 | 10,571 | 51,734 | 26,413 | 548 |
